# Intraclutch eggshell colour variation in birds: are females able to identify their eggs individually?

**DOI:** 10.7717/peerj.3707

**Published:** 2017-08-31

**Authors:** Miroslav Poláček, Michaela Bartíková, Herbert Hoi

**Affiliations:** 1 Slovak Academy of Sciences, Institute of Zoology, Bratislava, Slovakia; 2 Konrad Lorenz Institute of Ethology, Department of Integrative Biology and Evolution, University of Veterinary Medicine, Vienna, Austria

**Keywords:** Differential allocation, Eggshell colouration, Intraclutch variation, Sexual selection

## Abstract

**Background:**

One possibility suggested regarding female post-mating strategies is differential allocation into offspring investment. Female birds produce not only the largest, but also most colourful eggs of all oviparous taxa. Larger eggs provide space for bigger embryos, or more nutrition for their development, but the question why eggs are more colourful and why there is variation in eggshell colouration remains. In this context, the focus of interest has been to explain inter-clutch variation but in many bird species, eggshell colouration also varies within a clutch. Surprisingly, less attention has been paid to this phenomenon. Therefore, we propose the “female egg recognition” hypothesis, suggesting that mothers use colour characteristics to interpret egg attributes and allocate further investment into each egg accordingly. To evaluate the feasibility of the hypothesis, we tested several underlying predictions and examined their suitability using a dataset from our tree sparrow (*Passer montanus*) study. We predict (i) substantial within-clutch variation in eggshell colouration which, (ii) should be related to laying sequence, (iii) reflect egg quality and, (iv) should stimulate a female response.

**Methods:**

Eggshell coloration data were obtained via digital photography under standardized conditions, taken after clutch completion. Lightness (L*), representing the achromatic properties of an egg has been chosen as the most important predictor in dark cavities and was related to egg quality and position in the nest.

**Results:**

In our tree sparrows, first and mainly last eggs were less pigmented, providing information about laying order. Egg volume, which predicts chick quality, positively correlates with eggshell coloration. Finally, we could show that female tree sparrows placed darker, but not bigger, eggs into more central incubation positions.

**Discussion:**

All basic prerequisites for the “female egg recognition” hypothesis are fulfilled. In this context practicability and feasibility of the hypothesis and alternative explanations are discussed. However future work is necessary to determine a direct effect on offspring condition.

## Introduction

Colourful eggs are an exceptional feature of birds (Hauber, 2014). In this context, a flourishing number of hypotheses have been derived to explain this feature. There are several hypotheses suggesting a general function of eggshell colouration in relation to natural selection, including a cryptic function to avoid predation (Kilner, 2006; Wallace, 1889); a structural role, e.g., to strengthen the eggshell (Gosler, Higham & Reynolds, 2005) or facilitate thermoregulation (Bakken et al., 1978; Westmoreland, Schmitz & Burns, 2007) and disease resistance, via increasing antibacterial defence (Ishikawa et al., 2010).

Other hypotheses try to explain the function of egg colouration in relation to inter-clutch variation. Egg colour can serve to recognize own eggs (Birkhead, 1978) or eggs of brood parasites (Davies & Brooke, 1989). One function is related to sexual selection suggesting egg colour signalling female or offspring quality to manipulate male parental investment (Moreno & Osorno, 2003). In this case, eggshell colour information is addressed towards the male partner.

Finally, functions may be also related to intra-clutch variation in eggshell colour, which seems to be common in many bird species. Variation within a clutch can be manifested as a gradual decrease (Kendeigh, Kramer & Hamerstrom, 1956; Krist & Grim, 2007) or increase (Hargitai, Herényi & Török, 2008; Siefferman, Navara & Hill, 2006; Wink, Ristow & Wink, 1985) in pigment intensity with laying order. Sometimes, changes in eggshell colouration can be sudden, e.g., only the last-laid eggs are different. Odd-looking last-laid eggs were reported for many passerines, e.g., tree sparrows (*Passer montanus*) (Summers-Smith, 1995), house sparrows (*Passer domesticus*) (Lowther, 1988), reed warblers (*Acrocephalus scirpaceus*) (Davies & Brooke, 1988), as well as non-passerines, e.g., laughing gulls (*Larus atricapilla*) or common terns (*Sterna hirundo*) (Preston, 1957). In some species, like in northwestern crows (*Corvus caurinus*), not only the last, but also the first egg in a laying sequence can be paler (Verbeek, 1990).

In the case of a gradual decrease with egg laying, a non-adaptive mechanistic explanation, namely pigment depletion, would be sufficient to describe this phenomenon (Lowther, 1988) and may not necessarily imply a signalling function. Brood parasitism, in contrast, is known as an evolutionary force to reduce intraclutch variation in the host, because it increases the ability to identify parasitic eggs (Davies & Brooke, 1989; Øien, Moksnes & Røskaft, 1995). Finally, Yom-Tov (1980) proposes that pale last-laid eggs signal clutch completion to brood parasites and, consequently, protect hosts from costs connected to incubating parasitic eggs and the parasites to waste eggs.

Some hypotheses try to explain intraclutch eggshell variation in relation to predation. Verbeek (1990) proposes that paler last-laid eggs in a clutch are of lower reproductive value. In the case of partial predation, they may draw the attention of a predator and, thus, are sacrificed for the good of the clutch. Alternatively, various studies (Hockey, 1982; Lloyd et al., 2000) suggest that higher intraclutch variation may also enhance clutch crypsis, because more similar objects in the nest may be more visible. This may be true for species for which camouflage is very important, like for waders laying their eggs on the ground. Finally, another explanation in line with predation assumes that pigment production is costly for a laying female, and she can save on the last egg, because clutch attendance after its completion increases (Ruxton, Broom & Colegrave, 2001). However, this explanation is restricted to species for which colour variation affects only the last egg.

### The “female egg recognition” hypothesis

Here, we suggest an alternative, not mutually exclusive, hypothesis, which we term the “female egg recognition” hypothesis, in which the receiver of the eggshell colour information is the mother. Different to the sexual selected eggshell colour hypothesis (SSEC), which tries to explain the evolution of colourful eggs in relation to males and, in particular, variation between clutches (Moreno & Osorno, 2003), our hypothesis concentrates on within-clutch variation and, consequently, female ability to differentiate individual eggs via eggshell colouration.

Why should a mother want to individually identify her eggs, or at least their quality? Females should, in general try to increase their overall inclusive fitness (Dawkins, 1978). However, to maximize fitness females are also known to differentially allocate maternal resources into eggs within a clutch providing everything necessary for the embryo to develop (Mousseau & Fox, 1998). For example, androgens (Groothuis & Schwabl, 2002), corticosterone (Love et al., 2008), carotenoids (Török et al., 2007), immunoglobulins (Hargitai, Prechl & Török, 2006), and egg size in general (Slagsvold et al., 1984) vary with laying sequence. In line with this, we propose that a female may benefit by identifying individual eggs in her clutch because she can adjust post-laying investment more precisely to each or specific eggs. The main investment that can be made to the egg after it is laid is to incubate it. Incubation happens to the whole clutch at the same time, but the female can determine the position of the eggs e.g., putting them into the centre or the edge. Assuming that egg position in a clutch influences the thermal conditions (Caldwell & Cornwell, 1975; Huggins, 1941) one may expect fitness consequences, mainly via affecting growth rate (DuRant et al., 2013). This seems very fine scale investment, but it is known that early embryo conditions including also the effect of incubation can have significant long-term effects on an individual (Mousseau & Fox, 1998).

Thus for the hypothesis to be fulfilled one would ultimately predict that adjustments in investment translates into fitness/survival differences in the offspring. However, recognizable benefits of differential incubation treatment by females may vary and could be especially or only important under extreme environmental conditions. For instance, to increase overall fitness under harsh or optimal foraging conditions, females could prefer to invest more in high quality offspring. Alternatively they could also invest in low quality offspring to compensate for initial deficiencies and give them a better start (Darolová, Krištofík & Hoi, 2009). In Lesser grey shrikes, for instance, food availability seems to change during the incubation period in the so the called cockchafer (*Melolontha melolontha*) outbreak years (every third year). During incubation, mediated by a decrease in food availability, namely cockchafer, hatching success also decreased (Hoi et al., 2004). One possible explanation would be that females control the number of nestlings to be fed already during incubation. Thus the importance of the information gathered via eggshell colour may vary between breeding seasons, depending on environmental circumstances. In conclusion the ultimate prediction does not necessarily have to be fulfilled every year and also the extent of egg trait variation may vary between years.

Here we suggest that if eggs are of different value, eggshell colour can signal information about the quality of an egg (Krištofík et al., 2013; Krištofík et al., 2014), which may allow the female to treat eggs accordingly.

Our hypothesis applies to any form of within-clutch variation in eggshell colouration e.g., sudden or gradual changes, as long as it reflects laying order and/or variation in egg quality. The “female egg recognition” hypothesis, furthermore, does not assume a relationship between female quality and egg quality, which seems to be the case in some study systems (Moreno et al., 2005; Siefferman, Navara & Hill, 2006).

**Figure 1:**
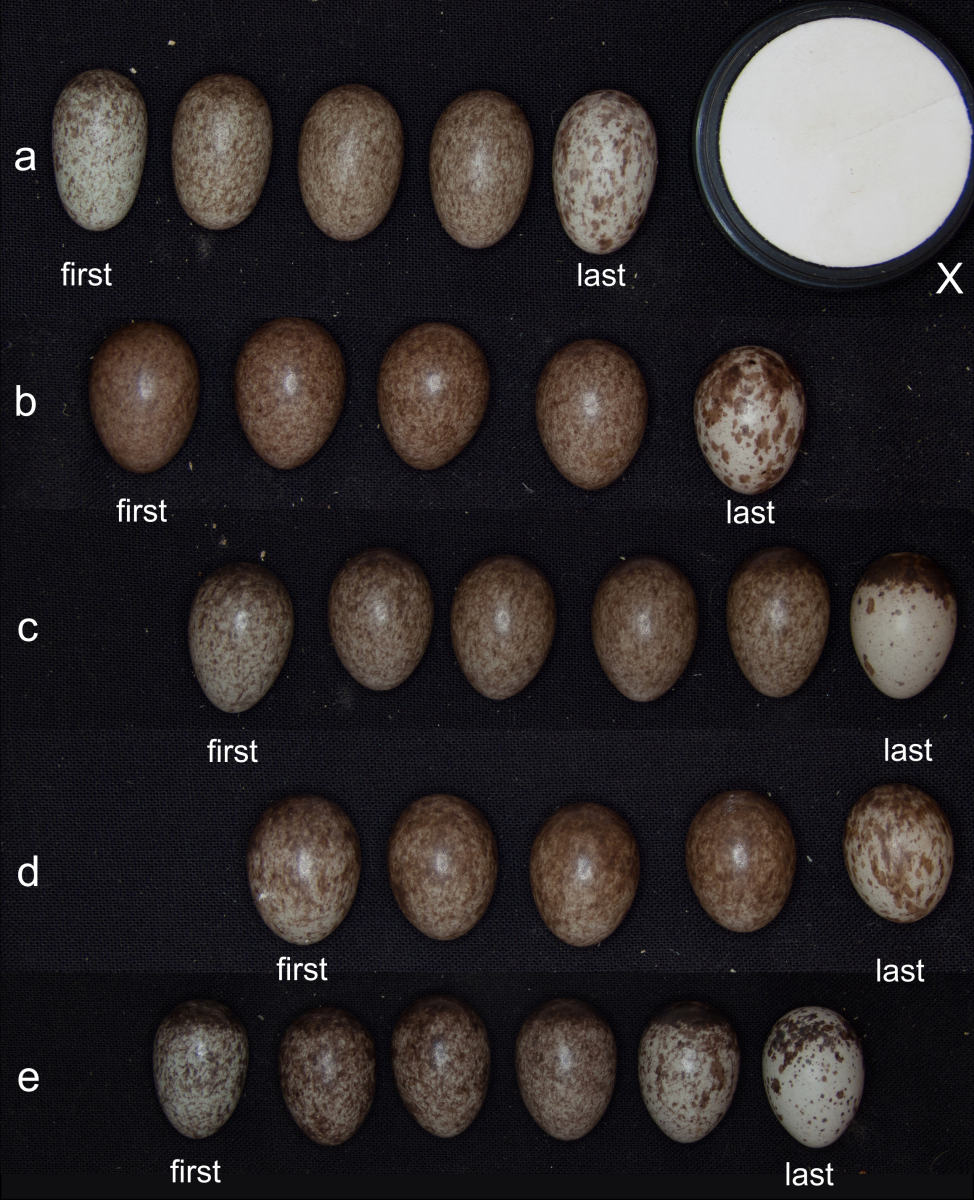
Intraclutch colour variation of the studied Tree sparrow population. Five examples (A–E) of a clutch of different females. The first and last egg of each clutch are indicated. Clutches were photographed in a wooden box on a black background and illuminated by a ring flash together with the White standard (Top Sensor Systems WS-2) (X) (photo by Miroslav Poláček).

To identify individual eggs females could use a variety of colour variables or other egg characteristics like egg size, volume or shape (see Fig. 1). The nest cavity of tree sparrows however, provides only very dim light conditions, the eggs touch and cover each other partly and/or being partially covered also by the nest material hanging from the nest rim. In such a situation it might be much more difficult to determine slight differences in egg length, which is difficult even when eggs are arranged in a row (Fig. 1). Egg volume is even more difficult to determine given that only the egg surface (clutch surface) can be seen and hence is only from above visible to the birds, in particular when breeding in deep narrow nest cavities like sparrows do.

For some species spottiness or egg colour patterns might be a possibility, but in this study we decided to use an achromatic parameter, namely lightness, as achromatic differences might be easier to capture by a bird’s eye in the dim light conditions of a nestbox.

Additionally, in a previous study, we found that lightness correlates with the amount of both important pigments in our study population (Poláček et al., 2017) and which are known to be indicative for egg quality in other species (Krist, 2011; Krištofík et al., 2013).

For this hypothesis to work, several preconditions have to be met. It would require (i) the existence of a substantial phenotypic within-clutch variation in some egg traits e.g., eggshell colouration, which, (ii) should be related to laying sequence, and (iii) should be linked to variation in offspring quality/fitness e.g., be reflected in egg quality, and (iv) to demonstrate that female adjust their investment (e.g., incubation position) according to variation in the egg trait.

In this context, we examine the potential of eggshell colouration to signal laying order and relative quality (e.g., egg volume) of eggs within a clutch. Furthermore, we explore a potential relationship between colouration of eggs and their position in a nest during incubation, which would indicate whether a female is treating eggs differently based on their colour.

In support of our hypothesis, a study on mallards (*Anas platyrhynchos*) found egg pigmentation and carotenoid concentration to decrease within a clutch, whereas egg and yolk size increase (Butler & McGraw, 2013). Verbeek (1990) also found that the last egg in a clutch had a decreased hatching success. One possible explanation for that could be differential egg treatment during incubation.

In this study, we aim to present the hypothesis and examine whether the preconditions of the “female egg recognition” hypothesis would be met and try to evaluate whether there is empirical support for it. For this purpose, we used a dataset from our tree sparrow (*Passer montanus*) population, with evidence for a high inter- and intraclutch egg colour variation (Poláček et al., 2013). Given that we use already available data, the results presented are only a first look into the predictions of the hypothesis.

## Methods

### Ethics statement

The study was done under the permission of the Nature Conservation Department of Lower Austria (RU5-BE-7/010-2011). The methods were carried out in accordance with the approved guidelines and all the experimental protocols were approved by the ethical commission of the Austrian Ministry of Sciences (BMWF-68.205/0245-II/3b/2012).

The study was conducted on private vineyards where we had permission to work.

### Study Species and Fieldwork

The research was performed in an agricultural landscape of Lower Austria, in the vicinity of the village Feuersbrunn (48°26′N, 15°47′E). Our study population was breeding in nest boxes installed in vineyards (288 in 2010, 296 in 2011), and fence posts and trees in an apricot orchard (36 in 2010, 43 in 2011). Pairs usually had three broods per year with an average of 5.1 ± 0.05 (mean ± SE) eggs. Nest building started in the first week of April. Hence, starting at the beginning of April, all nest boxes were checked weekly. To determine laying order, occupied nest boxes (with nest material) were then checked daily until the last egg of the clutch had been laid. Each egg was marked with a permanent marker at the day it was laid. In order to get an idea about female incubation behaviour, egg position in the clutch was monitored. Nest boxes were visited several times (5 − 11 times) during incubation, and the relative position of eggs to each other was sketched.

Eggshell coloration data were obtained using digital photography, using Fuji Finepix S200-EXR (aperture: 5.6, shutter speed: 1/1,000, ISO: 100). Digital pictures of clutches were taken after the beginning of incubation when the clutch was completed. Clutches were photographed in standardized conditions inside a wooden box illuminated by a ring flash on a black background, and white standard (Top Sensor Systems WS-2) was always included. Pictures were saved as RAW files and were processed in Adobe Photoshop. CIE Lab colour space was used for colour measurements. For each egg, four measurements were taken from randomly chosen places evenly distributed on the egg. These four samples covered together 10,404 pixels and on average 9% of the egg surface area on the picture. The average of these measurements was further used to analyse lightness (L*), which represents the achromatic properties of an object, ranging from 0 (black) to 100 (white). In a previous study we found that this colour measurement correlates with the amount of both eggshell pigments (biliverdin and protoporphyrin) in the studied population whereby protoporphyrin makes up a much bigger portion of egg pigments (Poláček et al., 2017).

As a proxy for egg quality we determined egg size (Krištofík et al., 2013). Therefore we measured the egg width and length and derived egg volume from these measurements according to Hoyt (1979).

### Statistical analyses

**Figure 2:**
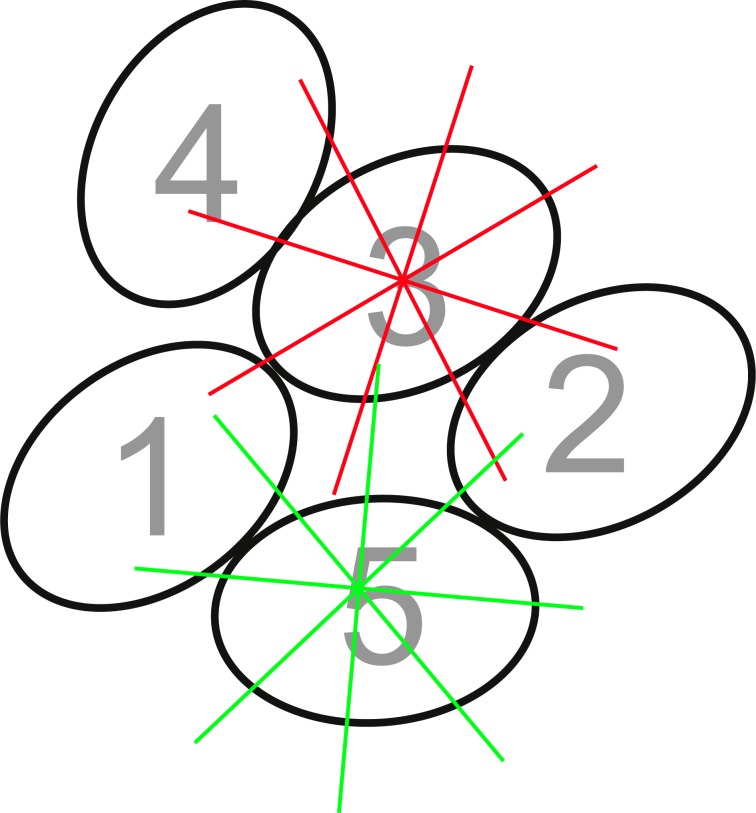
Example for a scheme, describing the method how egg position was determined in a clutch. For every egg we drew a line in eight directions from the centre of the egg. If a particular line crosses another egg it counts as 1, if not 0. Values for eight lines are then summed up for the egg. Thus the value for a single egg in a clutch could range from 0 up to 8 (when an egg is completely surrounded by other eggs). For example egg number 3 with red lines has a value of five, egg number 5 with green lines has a value of four.

Egg position was evaluated based on the relative central position in a clutch expressed in terms of in how many directions a particular egg has neighbouring eggs. For every egg we drew a line in eight directions from the centre of the egg and therefore values can vary from 0 to 8 (see Fig. 2). Values for each egg from different days were averaged.

Since our study focus was on intraclutch variation in eggshell colouration, between-clutch variation was removed by subtracting the mean of the clutch from the value, centring (Krist et al., 2004) for three variables—egg position, lightness, and egg volume. For this analysis, a linear mixed-effect model (LMM) in the R statistical program version 3.0.2 (Bates et al., 2014) was used. Package ‘lmerTest’ (Kuznetsova, Brockhoff & Christensen, 2013) was used to obtain degrees of freedom and *p*-values. Values reported in the results are for the final models. These were selected by backward stepwise elimination of non-significant terms from the initial model. Egg position was the dependent variable in this model, while lightness, egg volume, number of eggs, and laying order were independent variables. Nest box was included as a random effect. The origin of egg (parasitic or host) was included as random factor because nine nests had also been involved in a different experiment. When the nine nests were removed from analysis, the effect of eggshell colour on egg position remained significant (result not shown), thus we decided to retain the results with the higher sample size. The model was based on 157 eggs of 28 nests.

Similarly, centred data were also used to determine the relationship between lightness, egg volume, and laying order. An LMM was built with lightness as the dependent variable and egg volume and laying order as independent variables. Year and nest box were used as random factors. Data from the first brood in 2010 and 2011 were used, altogether 331 eggs of 66 nests.

All tests were two-tailed, and *p*-values smaller than 0.05 were considered to be statistically significant. When distribution of data violated normality and tests required normal distribution, data were log or square root transformed.

To be sure that the results of these models were not biased by a correlation between variables, we further calculated the variance inflation factors (VIF). None of the variables exceeded 2, which is the recommended threshold (Zuur, Ieno & Elphick, 2010).

## Results

**Figure 3:**
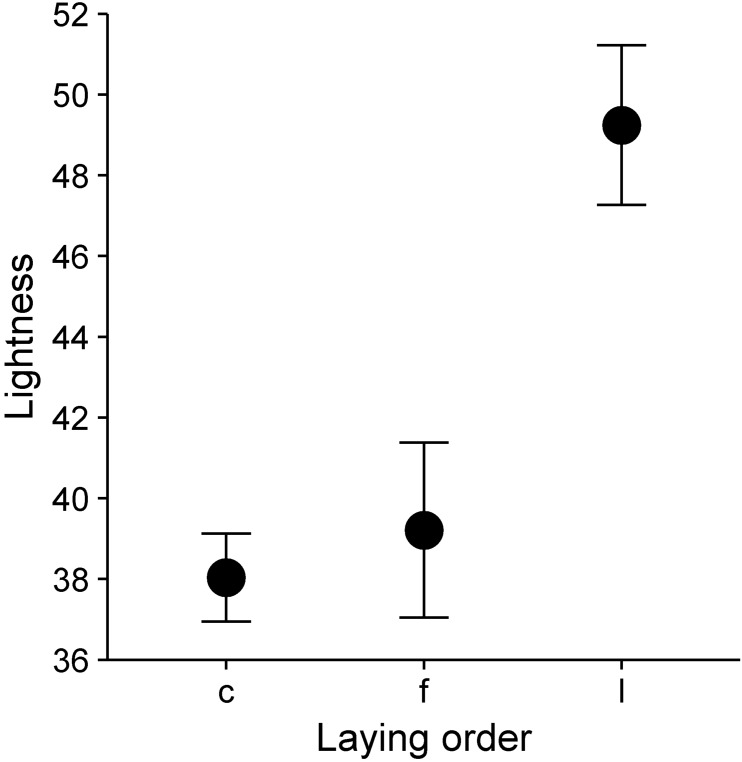
Plot showing differences in mean lightness (ranging from 0—black to 100—white) between eggs in central (c), first (f), and last (l) laying positions. Error bars represent 95% confidence intervals of the mean.

Eggshell colouration varies systematically with laying order within a clutch (Table 1, Fig. 3). The first (*df* = 326.9, *t* = 3.06, *p* = 0.002) and the last (*df* = 326.9, *t* = 21.91, *p* < 0.001) egg in the laying sequence were significantly paler than eggs in the middle of the laying sequence (Fig. 3). Egg volume was significantly negatively related to lightness (Table 1).

**Table 1:** Results of initial LMM models testing for effect of lightness (ranging from 0—black to 100—white) on egg volume and laying order of 331 eggs of 66 nests. Nest box and year were used as random effects. The variables retained in the final models are indicated by boldface.

Lightness	*Df*	*F*	B ± SE	*P*
**Egg volume**	**1,326.93**	**5.38**	−**0.001** ± **0.000**	**0.021**
**Laying order**	**2,326.93**	**243.49**		** <0.0001**

Egg lightness was the only significant variable explaining egg position during incubation (*df* = 1, 154.96, *F* = 7.85, *B* ± *SE* =  − 0.125 ± 0.045, *p* = 0.006, Table 2). Darker eggs in a clutch seem to be more frequently placed in a central position during incubation.

**Table 2:** Results of initial LMM model testing for effect of egg position on clutch features (157 eggs of 28 nests). Nest box was used as a random effect. The variables retained in the final models are indicated by boldface.

Egg position	*Df*	*F*	B ± SE	*P*
**Lightness**	**1,149.97**	**7.23**	−**0.161** ± **0.06**	**0.008**
Egg volume	1,149.97	1.52	0.000 ± 0.000	0.220
Number of eggs	1,149.97	0.04	−0.010 ± 0.049	0.842
Laying order	2,149.97	1.15		0.320
Parasitic egg	1,149.97	1.85		0.175

All variance inflation factors (see methods) were smaller than 1.83, which means that, even when following more conservative criteria, there should not be a problem of collinearity in the model.

## Discussion

In our study, eggshell colour substantially varied within clutches. This variation seems to be systematic, with the first and mainly last eggs being significantly less pigmented than the rest of the clutch (see Figs. 1 and 3). Consequently, eggshell colouration does provide information about laying order. We further found that egg volume, a predictor of egg/chick quality (Darolová, Krištofík & Hoi, 2014; Krist, 2011), positively correlates with eggshell colouration within a clutch. Finally, we could show that female tree sparrows placed mainly darker, but not necessarily bigger, eggs into a more central incubation position. Thus, basic preconditions necessary for the “female egg recognition” hypothesis to work are fulfilled and could be used to explain intraclutch variation in tree sparrows. However one study year namely 2010 was extremely harsh regarding food availability. So the extreme environmental circumstances could have very likely influenced the outcome of the results. Females may more likely differentially allocate into incubation investment in favour of high quality eggs when food availability is scarce and the chances for all nestlings to die would increase. The fact that females seem to use egg colour to identify individual eggs does not rule out other functions.

Regarding alternative hypotheses, we can exclude pigment depletion to explain intraclutch colour variation (see Introduction), because in our model species the change in colouration is not gradual, and it is the first and the last eggs that are paler than eggs in the middle of the laying sequence. Furthermore, hypotheses associated with predation are probably less important to explain intraclutch colour variation, because clutches of the cavity breeding tree sparrows are rarely predated by visually-oriented predators (own observations). Intraspecific brood parasitism could be potentially important, but in a previous study, we did not find strong evidence for intraspecific brood parasitism in our tree sparrow population (Poláček et al., 2013). However, this does not exclude its role for the evolution of within-clutch variation in eggshell colouration. Yom-Tov (1980) proposes that pale last-laid eggs advertise to brood parasites that a clutch is complete, and eggs laid thereafter are not likely to hatch. Such a signalling function would be evolutionary stable and resistant to cheating only under strict conditions, namely, costly production of pale eggs early in the clutch and production of the last pale eggs that are not too costly, compared to the cost of parasitism (Ruxton, Broom & Colegrave, 2001).

An important precondition for females to use egg shell colouration to manipulate hatching order is that temperature in the nest decreases from the centre to the edge. This was indeed documented in chipping sparrows (*Spizella passerine*), for which a maximal temperature difference is 4.6 °C between two eggs of the same clutch, and in mallards, with a temperature difference of 12.2 °C (Caldwell & Cornwell, 1975; Huggins, 1941). In great tits (*Parus major*), eggs in the outer incubation position were cooler than eggs in the centre and also cool quicker after the female left to forage (Boulton & Cassey, 2012). Furthermore, it was found that even slight differences in incubation temperature within a range of natural incubation may have strong effects on offspring development and quality (DuRant et al., 2013). Offspring might develop slower at lower temperatures, and may suffer lower hatching or fledging success, reduced body condition, immunocompetence, post-hatching growth rates, locomotory performance, higher baseline and stress-induced corticosterone concentrations, and even lower reproductive success in their first breeding season (DuRant et al., 2010; DuRant et al., 2013; Hepp & Kennamer, 2012; Hepp, Kennamer & Johnson, 2006; Hopkins et al., 2011; Martin et al., 2007).

Incubating females may adjust egg position to manipulate the level of hatching asynchrony. If the only egg quality that colouration signals is its order in laying sequence, it would be enough for the “female egg recognition” hypothesis to work. For example, in species that start incubation with the penultimate egg, it might be beneficial to know exactly which egg was laid after the beginning of incubation and, thus, is behind with development. This information might then be used in both ways, to increase or decrease hatching asynchrony. Tree sparrows normally start to incubate with the penultimate egg. In a clutch with five eggs, for example, hatching asynchrony produces differences in chick age of up to three days (Summers-Smith, 1995). Under unfavourable conditions, this may cause brood reduction, because bigger differences in chick age may lower the competitive ability of younger siblings. Using egg information via colouration, mothers, in contrast, could even do the opposite, namely create more equal conditions by favouring weaker individuals. In bearded tits, it was found that female offspring hatch first, which improves their early developmental conditions in comparison to the faster growing, but later hatching, male offspring (Darolová, Krištofík & Hoi, 2009). Thus, selective incubation may be a possibility to influence offspring in diverse directions.

As predicted by theoretical models, natural selection should favour parental ability to recognise offspring quality, as different fitness rewards may result from allocation of the same amount of resources to offspring differing in quality (Haig, 1990). Given that differential allocation of maternal egg investment is quite common (Mousseau & Fox, 1998), and that bird species show clear preferences of, for example, certain offspring in the nest according to their quality (Saino et al., 2000), it is likely that females may apply differential allocation rules already during the incubation period. In this context, the ability to recognize individual eggs may allow females to favour specific eggs, for example, by adjusting their incubation conditions.

There are a few studies also linking colour variation within a clutch to parameters of egg quality other than order in laying sequence. In mallards, Butler & McGraw (2013) revealed that both eggshell pigmentation and carotenoid concentration decreased and egg and yolk size increased with laying order. Furthermore, Verbeek (1990) found in northwestern crows that paler last-laid eggs even had significantly reduced hatching success. In a more subtle way, differential allocation during incubation may influence hatching order, level of hatching asynchrony, or nestling condition at hatching, e.g., to support more valuable nestlings getting a better start, which can be an important fitness determinant (Darolová, Krištofík & Hoi, 2009). There are only a few studies showing that birds might actively adjust incubation position to discriminate in favour of certain eggs in their clutch. In American coots, parasitic eggs are placed on the periphery of a clutch. Consequently, eggs on the periphery hatched later than eggs in the centre of a clutch (Lyon, 2003). Furthermore, Caldwell & Cornwell (1975) noticed a non-random egg position of mallard clutches, as some eggs are in the middle of the clutch more often than others. They suggested that, because of the shape of the nest bowl, heavier eggs tend to be in the centre more often. However, in our model, all VIF were smaller than 1.83 (see Results), which means that there was no problem of collinearity, even when following more conservative criteria (Zuur, Ieno & Elphick, 2010). This consequently suggests that, in our study, egg volume was not significantly related to position.

As already mentioned, another advantage of the “female egg recognition” hypothesis is that it can be applied to any type of intraclutch variation. As long as eggshell colour reflects laying order and/or egg quality, it is independent of how many eggs (at least one) of a clutch are involved and whether there is a gradual change, for example, with laying order or a sudden change producing diverging egg colour categories.

From our study, we unfortunately lack data on female fitness consequences to support the hypothesis. One possibility in that direction would be to determine offspring quality in relation to egg colour; however we did not do this yet, since it is difficult to identify which nestling originates from which egg. Thus, to test the general applicability of the “female egg recognition” hypothesis, it would be necessary, for example, to examine how frequently differential incubation investment may occur and what consequences (offspring quality, survival) they may have. No study we are aware of has paid attention to temperature gradients within a clutch and possible functions, e.g., in relation to bacterial defence, hatching synchronisation, or offspring phenotype.

In conclusion, our results suggest that preconditions in support of the “female egg recognition” hypothesis are fulfilled. However, more detailed research would be necessary to evaluate (i) whether this hypothesis may substantially broaden our understanding of adaptive egg characteristics and (ii) its general applicability.

##  Supplemental Information

10.7717/peerj.3707/supp-1 Supplemental Information 1 Analysis of egg position and egg volume

## Additional Information and Declarations

### Field Study Permissions

The following information was supplied relating to field study approvals (i.e., approving body and any reference numbers): The study was done under the permission of the Nature Conservation Department of Lower Austria (RU5-BE-7/010-2011).

### Data Availability

The following information was supplied regarding data availability: The raw data has been supplied as a Supplementary File.

## References

[ref-1] Bakken GS, Vanderbilt VC, Buttemer WA, Dawson WR (1978). Avian eggs: thermoregulatory value of very high near-infrared reflectance. Science.

[ref-2] Bates D, Mächler M, Bolker B, Walker S (2014). lme4: linear mixed-effects models using Eigen and S4. http://CRAN.R-project.org/package=lme4.

[ref-3] Birkhead TR (1978). Behavioral adaptations to high density nesting in Common Guillemot *Uria aalge*. Animal Behaviour.

[ref-4] Boulton RL, Cassey P (2012). How avian incubation behaviour influences egg surface temperatures: relationships with egg position, development and clutch size. Journal of Avian Biology.

[ref-5] Butler MW, McGraw KJ (2013). Eggshell coloration reflects both yolk characteristics and dietary carotenoid history of female mallards. Functional Ecology.

[ref-6] Caldwell PJ, Cornwell GW (1975). Incubation behavior and temperatures of the mallard duck. The Auk.

[ref-7] Darolová A, Krištofík J, Hoi H (2009). Extreme brood sex ratios in Bearded Tits *Panurus biarmicus*. Ibis.

[ref-8] Darolová A, Krištofík J, Hoi H (2014). Vegetation type variation in marsh habitats: does it affect nest site selection, reproductive success, and maternal investment in Reed Warblers?. Journal of Ornithology.

[ref-9] Davies NB, Brooke MDL (1988). Cuckoos versus reed warblers: adaptations and counteradaptations. Animal Behaviour.

[ref-10] Davies NB, Brooke MDL (1989). An experimental study of co-evolution between the cuckoo, *Cuculus canorus*, and its hosts. I. Host egg discrimination. Journal of Animal Ecology.

[ref-11] Dawkins R (1978). The selfish gene.

[ref-12] DuRant SE, Hepp GR, Moore IT, Hopkins BC, Hopkins WA (2010). Slight differences in incubation temperature affect early growth and stress endocrinology of wood duck (*Aix sponsa*) ducklings. Journal of Experimental Biology.

[ref-13] DuRant SE, Hopkins WA, Hepp GR, Walters JR (2013). Ecological, evolutionary, and conservation implications of incubation temperature-dependent phenotypes in birds. Biological Reviews.

[ref-14] Gosler AG, Higham JP, Reynolds SJ (2005). Why are birds’ eggs speckled?. Ecology Letters.

[ref-15] Groothuis TG, Schwabl H (2002). Determinants of within- and among-clutch variation in levels of maternal hormones in Black-Headed Gull eggs. Functional Ecology.

[ref-16] Haig D (1990). Brood reduction and optimal parental investment when offspring differ in quality. American Naturalist.

[ref-17] Hargitai R, Herényi M, Török J (2008). Eggshell coloration in relation to male ornamentation, female condition and egg quality in the collared flycatcher *Ficedula albicollis*. Journal of Avian Biology.

[ref-18] Hargitai R, Prechl J, Török J (2006). Maternal immunoglobulin concentration in Collared Flycatcher (*Ficedula albicollis*) eggs in relation to parental quality and laying order. Functional Ecology.

[ref-19] Hauber ME (2014). The book of eggs. A lifesize guide to the eggs of six hundred of the world’s bird species.

[ref-20] Hepp GR, Kennamer RA (2012). Warm is better: incubation temperature influences apparent survival and recruitment of wood ducks (*Aix sponsa*). PLOS ONE.

[ref-21] Hepp GR, Kennamer RA, Johnson MH (2006). Maternal effects in Wood Ducks: incubation temperature influences incubation period and neonate phenotype. Functional Ecology.

[ref-22] Hockey PAR (1982). Adaptiveness of nest site selection and egg coloration in the African black oystercatcher *Haematopus moquini*. Behavioral Ecology and Sociobiology.

[ref-23] Hoi H, Krištín A, Valera F, Hoi C (2004). Clutch enlargement in lesser gray shrikes (*Lanius minor*) in Slovakia when food is superabundant: a maladaptive response?. The Auk.

[ref-24] Hopkins BC, DuRant SE, Hepp GR, Hopkins WA (2011). Incubation temperature influences locomotor performance in young wood ducks (*Aix sponsa*). Journal of Experimental Zoology Part A: Ecological Genetics and Physiology.

[ref-25] Hoyt DF (1979). Practical methods of estimating volume and fresh weight of bird eggs. The Auk.

[ref-26] Huggins RA (1941). Egg temperatures of wild birds under natural conditions. Ecology.

[ref-27] Ishikawa S, Suzuki K, Fukuda E, Arihara K, Yamamoto Y, Mukai T, Itoh M (2010). Photodynamic antimicrobial activity of avian eggshell pigments. FEBS Letters.

[ref-28] Kendeigh SC, Kramer TC, Hamerstrom F (1956). Variations in egg characteristics of the house wren. The Auk.

[ref-29] Kilner RM (2006). The evolution of egg colour and patterning in birds. Biological Reviews.

[ref-30] Krist M (2011). Egg size and offspring quality: a meta-analysis in birds. Biological Reviews.

[ref-31] Krist M, Grim T (2007). Are blue eggs a sexually selected signal of female collared flycatchers? A cross-fostering experiment. Behavioral Ecology and Sociobiology.

[ref-32] Krist M, Remeš V, Uvírová L, Nádvornik P, Bureš S (2004). Egg size and offspring performance in the collared flycatcher (*Ficedula albicollis*): a within-clutch approach. Oecologia.

[ref-33] Krištofík J, Darolová A, Griggio M, Majtán J, Okuliarová M, Zeman M, Zídková L, Hoi H (2013). Does egg colouration signal female and egg quality in reed warbler (*Acrocephalus scirpaceus*)?. Ethology Ecology & Evolution.

[ref-34] Krištofík J, Darolová A, Majtan J, Okuliarová M, Zeman M, Hoi H (2014). Do females invest more into eggs when males sing more attractively? Postmating sexual selection strategies in a monogamous reed passerine. Ecology and Evolution.

[ref-35] Kuznetsova A, Brockhoff PB, Christensen RHB (2013). lmerTest: tests for random and fixed effects for linear mixed effect models (lmer objects of lme4 package). http://CRAN.R-project.org/package=lmerTest.

[ref-36] Lloyd P, Plagányi É, Lepage D, Little RM, Crowe TM (2000). Nest-site selection, egg pigmentation and clutch predation in the ground-nesting Namaqua Sandgrouse *Pterocles namaqua*. Ibis.

[ref-37] Love OP, Wynne-Edwards KE, Bond L, Williams TD (2008). Determinants of within- and among-clutch variation in yolk corticosterone in the European starling. Hormones and Behavior.

[ref-38] Lowther PE (1988). Spotting pattern of the last laid egg of the house sparrow. Journal of Field Ornithology.

[ref-39] Lyon BE (2003). Egg recognition and counting reduce costs of avian conspecific brood parasitism. Nature.

[ref-40] Martin TE, Auer SK, Bassar RD, Niklison AM, Lloyd P (2007). Geographic variation in avian incubation periods and parental influences on embryonic temperature. Evolution.

[ref-41] Moreno J, Morales J, Lobato E, Merino S, Tomás G, Martínez-de la Puente J (2005). Evidence for the signaling function of egg color in the pied flycatcher *Ficedula hypoleuca*. Behavioral Ecology.

[ref-42] Moreno J, Osorno JL (2003). Avian egg colour and sexual selection: does eggshell pigmentation reflect female condition and genetic quality?. Ecology Letters.

[ref-43] Mousseau TA, Fox CW (1998). The adaptive significance of maternal effects. Trends in Ecology & Evolution.

[ref-44] Øien IJ, Moksnes A, Røskaft E (1995). Evolution of variation in egg color and marking pattern in European Passerines: adaptations in a coevolutionary arms race with the cuckoo, *Cuculus canorus*. Behavioral Ecology.

[ref-45] Poláček M, Griggio M, Bartíková M, Hoi H (2013). Nest sanitation as the evolutionary background for egg ejection behaviour and the role of motivation for object removal. PLOS ONE.

[ref-46] Poláček M, Griggio M, Mikšík I, Bartíková M, Eckenfellner M, Hoi H (2017). Eggshell coloration and its importance in postmating sexual selection. Ecology and Evolution.

[ref-47] Preston FW (1957). Pigmentation of eggs: variation in the clutch sequence. The Auk.

[ref-48] Ruxton GD, Broom M, Colegrave N (2001). Are unusually colored eggs a signal to potential conspecific brood parasites?. The American Naturalist.

[ref-49] Saino N, Ninni P, Incagli M, Calza S, Sacchi R, Møller AP (2000). Begging and parental care in relation to offspring need and condition in the barn swallow (*Hirundo rustica*). The American Naturalist.

[ref-50] Siefferman L, Navara KJ, Hill GE (2006). Egg coloration is correlated with female condition in eastern bluebirds *(Sialia sialis*). Behavioral Ecology and Sociobiology.

[ref-51] Slagsvold T, Sandvik J, Rofstad G, Lorentsen Ö, Husby M (1984). On the adaptive value of intraclutch egg-size variation in birds. The Auk.

[ref-52] Summers-Smith JD (1995). The tree sparrow.

[ref-53] Török J, Hargitai R, Hegyi G, Matus Z, Michl G, Péczely P, Rosivall B, Tóth G (2007). Carotenoids in the egg yolks of collared flycatchers (*Ficedula albicollis*) in relation to parental quality, environmental factors and laying order. Behavioral Ecology and Sociobiology.

[ref-54] Verbeek NAM (1990). Differential predation on eggs in clutches of northwestern crows: the importance of egg color. The Condor.

[ref-55] Wallace AR (1889). Darwinism. An exposition of the theory of natural selection. With some of its applications.

[ref-56] Westmoreland D, Schmitz M, Burns KE (2007). Egg color as an adaptation for thermoregulation. Journal of Field Ornithology.

[ref-57] Wink M, Ristow D, Wink C (1985). Biology of eleonora’s falcon (*Falco eleonorae*): 7. Variability of clutch size, egg dimensions and egg coloring. Raptor Research.

[ref-58] Yom-Tov Y (1980). Intraspecific nest parasitism in birds. Biological Reviews.

[ref-59] Zuur AF, Ieno EN, Elphick CS (2010). A protocol for data exploration to avoid common statistical problems. Methods in Ecology and Evolution.

