# Supplementary material for: Intraclutch eggshell colour variation in birds: are females able to identify their eggs individually?
Source: PeerJ. 2017 Aug 31;5:e3707. doi: 10.7717/peerj.3707 (PMC5581944; doi:10.7717/peerj.3707)
Supplement: Supplemental Information 1 [file peerj-05-3707-s001.pdf]

box	clutch size	parasitic egg	laying order	orderFCL	position	centered position	lightness	centered lightness	egg volume	centered egg volume
D3	6	0	1	f	4,625	0,146	53	3,417	1571,751919	-10,550
D3	6	0	2	c	4,375	-0,104	46,75	-2,833	1654,422074	72,120
D3	6	0	3	c	4,250	-0,229	46	-3,583	1671,209501	88,908
D3	6	0	4	c	4,375	-0,104	45,75	-3,833	1618,963686	36,662
D3	6	0	5	c	4,625	0,146	47,5	-2,083	1574,972614	-7,329
D3	6	0	6	l	4,625	0,146	58,5	8,917	1402,490144	-179,812
I21	4	0	1	f	3,364	-0,318	33,75	-0,500	1975,181161	-29,448
I21	4	0	2	c	4,091	0,409	28,25	-6,000	2003,008876	-1,620
I21	4	0	3	c	3,727	0,045	29,25	-5,000	1994,980121	-9,649
I21	4	0	4	l	3,545	-0,137	45,75	11,500	2045,347031	40,718
I23	6	0	1	f	4,417	-0,139	61,25	0,083	1616,187173	-74,701
I23	6	0	2	c	5,583	1,027	48,25	-12,917	1738,010183	47,122
I23	6	0	3	c	4,167	-0,389	69,25	8,083	1776,187233	85,299
I23	6	0	4	c	4,250	-0,306	59,5	-1,667	1677,008076	-13,880
I23	6	0	5	c	3,917	-0,639	64,5	3,333	1672,154772	-18,733
I23	6	0	6	l	5,000	0,444	64,25	3,083	1665,779047	-25,109
I25	5	0	1	f	4,100	-0,060	41,25	-5,350	1977,506303	-2,287
I25	5	0	2	c	4,700	0,540	43,5	-3,100	1997,426031	17,633
I25	5	0	3	c	4,700	0,540	46,25	-0,350	1921,279581	-58,513
I25	5	0	4	c	3,300	-0,860	47,5	0,900	2011,25414	31,461
I25	5	0	5	l	4,000	-0,160	54,5	7,900	1991,498588	11,706
J29	5	0	1	f	3,455	-0,836	62,25	6,250	1732,214213	-195,071
J29	5	0	2	c	3,909	-0,382	52,75	-3,250	1877,178249	-50,107
J29	5	0	3	c	4,818	0,527	50,25	-5,750	1997,827434	70,542
J29	5	0	4	c	4,818	0,527	48,75	-7,250	1956,395904	29,110
J29	5	0	5	l	4,455	0,164	66	10,000	2072,812443	145,527
J29b	5	0	1	f	4,125	-0,075	43,25	-4,250	1898,353791	-126,766
J29b	5	0	2	c	3,750	-0,450	44,5	-3,000	1986,316515	-38,803
J29b	5	0	3	c	3,750	-0,450	44,75	-2,750	2013,791891	-11,328
J29b	5	0	4	c	4,250	0,050	47	-0,500	2086,124897	61,005
J29b	5	0	5	l	5,125	0,925	58	10,500	2141,010893	115,891
J4	5	0	1	f	4,455	0,200	41,5	-0,600	2181,841812	122,483
J4	5	0	2	c	4,000	-0,255	42	-0,100	2022,395785	-36,963
J4	5	0	3	c	4,818	0,563	39,25	-2,850	2030,249018	-29,109
J4	5	0	4	c	4,182	-0,073	37,75	-4,350	2061,372368	2,014
J4	5	0	5	l	3,818	-0,437	50	7,900	2000,933477	-58,425
J44	6	0	1	f	3,889	-0,518	31,25	-0,750	1768,808125	82,978
J44	6	0	2	c	4,556	0,149	23,75	-8,250	1655,127349	-30,703
J44	6	0	3	c	4,444	0,037	32,75	0,750	1611,412679	-74,418
J44	6	0	4	c	5,000	0,593	30	-2,000	1719,324352	33,494
J44	6	0	5	c	4,222	-0,185	25,5	-6,500	1669,249808	-16,581
J44	6	0	6	l	4,333	-0,074	48,75	16,750	1691,059944	5,230
K10	6	0	1	f	4,500	0,056	61,5	6,083	1923,478427	17,959
K10	6	0	2	c	4,000	-0,444	50,75	-4,667	1876,234816	-29,285
K10	6	0	3	c	4,833	0,389	52	-3,417	1965,481963	59,962
K10	6	0	4	c	4,750	0,306	52,5	-2,917	1987,752967	82,233
K10	6	0	5	l	4,416	-0,028	62,75	7,333	1918,148882	12,629
K10	6	1	2	c	4,167	-0,277	53	-2,417	1762,020776	-143,499
K6	6	0	1	f	4,800	0,033	44,25	0,750	2177,521741	134,906
K6	6	0	2	c	4,800	0,033	36	-7,500	2106,104621	63,489
K6	6	0	3	c	4,200	-0,567	40	-3,500	2034,100422	-8,515
K6	6	0	4	c	5,000	0,233	39,5	-4,000	2108,596265	65,981
K6	6	0	5	l	5,000	0,233	44,25	0,750	2065,407906	22,792
K6	6	1	2	l	4,800	0,033	57	13,500	1763,963218	-278,652
M2	7	0	1	f	4,500	-0,250	44,75	-2,500	1804,269664	-132,855
M2	7	0	2	c	5,125	0,375	42,5	-4,750	1955,248273	18,123
M2	7	0	3	c	4,625	-0,125	44,75	-2,500	2004,358635	67,233
M2	7	0	4	c	5,375	0,625	45	-2,250	1957,632801	20,508
M2	7	0	5	c	4,250	-0,500	46,25	-1,000	1947,295089	10,170
M2	7	0	6	l	4,875	0,125	63,25	16,000	1827,516909	-109,608
M2	7	1	2	l	4,500	-0,250	44,25	-3,000	2063,554719	126,430
O22	7	0	1	f	5,500	0,829	42,25	-3,000	1906,122634	-25,800
O22	7	0	2	c	4,200	-0,471	44,5	-0,750	1930,676879	-1,246
O22	7	0	3	c	4,700	0,029	40,75	-4,500	2010,1948	78,272
O22	7	0	4	c	4,200	-0,471	44,5	-0,750	2005,328573	73,406
O22	7	0	5	c	5,200	0,529	35	-10,250	1896,031159	-35,892
O22	7	0	6	l	4,300	-0,371	56,75	11,500	1802,267998	-129,655
O22	7	1	2	c	4,600	-0,071	53	7,750	1972,837423	40,915
O22b	5	0	1	f	4,125	-0,075	44,75	-3,550	2105,554176	-48,991
O22b	5	0	2	c	4,250	0,050	47,25	-1,050	2178,203753	23,659
O22b	5	0	3	c	4,124	-0,076	46,25	-2,050	2139,874181	-14,671
O22b	5	0	4	c	4,375	0,175	48,25	-0,050	2179,563383	25,018
O22b	5	0	5	l	4,125	-0,075	55	6,700	2169,530591	14,985

P20	6	0	1	f		4,128	-0,292	38,25	-7,750	1998,866858	102,098
P20	6	0	2	c		4,000	-0,420	47,5	1,500	1902,065743	5,297
P20	6	0	3	c		4,364	-0,056	46,5	0,500	1833,443166	-63,325
P20	6	0	4	c		5,000	0,580	43,75	-2,250	1766,073013	-130,696
P20	6	0	5	l		4,128	-0,292	48	2,000	1674,798612	-221,970
P20	6	1	2	f		4,900	0,480	52	6,000	2205,364141	308,596
P4	6	0	1	f		5,182	0,803	32,75	-5,458	2030,662188	28,386
P4	6	0	2	c		4,000	-0,379	30	-8,208	2038,217448	35,941
P4	6	0	3	c		3,364	-1,015	31,5	-6,708	2052,680034	50,404
P4	6	0	4	c		4,000	-0,379	36,25	-1,958	2049,255774	46,979
P4	6	0	5	l		5,364	0,985	43	4,792	2031,851215	29,575
P4	6	1	2	c		4,364	-0,015	55,75	17,542	1810,991314	-191,285
P8	5	0	1	f		3,857	-0,257	55,25	2,000	1819,832098	-29,101
P8	5	0	2	c		3,286	-0,828	55,5	2,250	1883,962848	35,030
P8	5	0	3	c		4,571	0,457	48	-5,250	1903,389197	54,456
P8	5	0	4	c		4,714	0,600	51	-2,250	1894,731933	45,799
P8	5	0	5	l		4,143	0,029	56,5	3,250	1742,750592	-106,183
P9	6	0	1	f		5,200	0,483	39	-6,833	2148,897154	38,897
P9	6	0	2	c		4,800	0,083	40,25	-5,583	2079,212239	-30,788
P9	6	0	3	c		5,100	0,383	38,75	-7,083	2181,923083	71,923
P9	6	0	4	c		4,900	0,183	43,5	-2,333	2232,117237	122,117
P9	6	0	5	l		3,700	-1,017	57,75	11,917	2187,761245	77,761
P9	6	1	2	c		4,600	-0,117	55,75	9,917	1830,0889	-279,911
R13	5	0	1	f		3,375	-0,775	55,5	1,500	1787,061587	-174,181
R13	5	0	2	c		4,375	0,225	52	-2,000	2051,948278	90,705
R13	5	0	3	c		4,625	0,475	61,25	7,250	2166,726363	205,483
R13	5	0	4	l		3,750	-0,400	59,25	5,250	1773,050014	-188,193
R13	5	1	2	c		4,625	0,475	42	-12,000	2027,4289	66,186
R9	7	0	1	f		5,375	0,821	48,5	5,464	2046,972975	-24,148
R9	7	0	2	c		4,250	-0,304	43,25	0,214	2034,349201	-36,772
R9	7	0	3	c		5,250	0,696	35,25	-7,786	2080,133067	9,012
R9	7	0	4	c		3,750	-0,804	38,75	-4,286	2320,310434	249,189
R9	7	0	5	c		4,500	-0,054	34,75	-8,286	2172,06329	100,942
R9	7	0	6	l		3,750	-0,804	54	10,964	2079,540606	8,419
R9	7	1	2		c	5,000	0,446	46,75	3,714	1764,480668	-306,641
V4	5	0	1	f		4,500	0,220	40,5	2,700	1905,339293	59,660
V4	5	0	2	c		3,900	-0,380	35	-2,800	1813,792723	-31,886
V4	5	0	3	c		5,100	0,820	30,75	-7,050	1852,493506	6,814
V4	5	0	4	c		4,300	0,020	34,75	-3,050	1841,432701	-4,246
V4	5	0	5	l		3,600	-0,680	48	10,200	1815,337556	-30,342
V9	6	0	1	f		4,900	0,333	35,75	0,667	1792,598463	60,659
V9	6	0	2	c		4,000	-0,567	31,25	-3,833	1733,385069	1,445
V9	6	0	3	c		4,700	0,133	31,25	-3,833	1656,980796	-74,959
V9	6	0	4	c		4,000	-0,567	32,5	-2,583	1751,699569	19,760
V9	6	0	5	c		4,800	0,233	35	-0,083	1756,186179	24,247
V9	6	0	6	l		5,000	0,433	44,75	9,667	1700,78756	-31,152
W2	5	0	1	f		4,875	0,725	50,5	5,650	2190,942672	217,899
W2	5	0	2	c		3,750	-0,400	43,75	-1,100	1962,436686	-10,607
W2	5	0	3	c		4,500	0,350	39,5	-5,350	1942,239805	-30,804
W2	5	0	4	c		3,750	-0,400	41,25	-3,600	1880,93021	-92,113
W2	5	0	5	l		3,875	-0,275	49,25	4,400	1888,668159	-84,375
W3	5	0	1	f		3,778	-0,311	50,75	6,450	1891,808354	19,360
W3	5	0	2	c		4,000	-0,089	45	0,700	1954,142928	81,694
W3	5	0	3	c		4,444	0,355	40	-4,300	1868,263416	-4,185
W3	5	0	4	c		4,000	-0,089	42,75	-1,550	1858,272998	-14,176
W3	5	0	5	l		4,222	0,133	43	-1,300	1789,756077	-82,693
W4	5	0	1	f		4,125	0,000	36,75	-2,050	1673,750041	-161,203
W4	5	0	2	c		3,875	-0,250	34,25	-4,550	1810,816502	-24,137
W4	5	0	3	c		4,750	0,625	35,75	-3,050	1941,228749	106,275
W4	5	0	4	c		4,375	0,250	36,25	-2,550	1895,332796	60,379
W4	5	0	5	l		3,500	-0,625	51	12,200	1853,63854	18,685
W5	6	0	1	f		5,600	1,167	48,25	0,500	1929,401719	27,618
W5	6	0	2	c		4,800	0,367	45,25	-2,500	1889,633405	-12,150
W5	6	0	3	c		4,200	-0,233	45	-2,750	1883,780793	-18,002
W5	6	0	4	c		3,400	-1,033	45,5	-2,250	1971,332368	69,549
W5	6	0	5	c		4,600	0,167	42,5	-5,250	1870,385214	-31,398
W5	6	0	6	l		4,000	-0,433	60	12,250	1866,166229	-35,617
W7	6	0	1	f		4,625	-0,021	32	-0,375	2015,513064	1,749
W7	6	0	2	c		4,625	-0,021	30,25	-2,125	1970,413568	-43,350
W7	6	0	3	c		5,125	0,479	29,5	-2,875	1977,2751	-36,489
W7	6	0	4	c		4,625	-0,021	35,25	2,875	2039,689071	25,925
W7	6	0	5	c		4,500	-0,146	29,5	-2,875	2028,758262	14,995
W7	6	0	6	l		4,375	-0,271	37,75	5,375	2050,933158	37,169
Z1	5	0	1	f		4,500	0,340	55,5	1,200	1567,238773	-172,100

Z1	5	0	2	c	4,200	0,040	50,75	-3,550	1759,986938	20,648
Z1	5	0	3	c	4,300	0,140	51,25	-3,050	1773,508472	34,170
Z1	5	0	4	c	4,100	-0,060	52,5	-1,800	1808,497512	69,159
Z1	5	0	5	l	3,700	-0,460	61,5	7,200	1787,462994	48,124
Z5	6	0	1	f	4,100	-0,417	42,5	-4,375	1920,44464	75,290
Z5	6	0	2	c	4,000	-0,517	44	-2,875	1785,521475	-59,633
Z5	6	0	3	c	5,250	0,733	43,25	-3,625	1857,248174	12,093
Z5	6	0	4	c	4,625	0,108	48,5	1,625	1844,570154	-0,585
Z5	6	0	5	c	4,625	0,108	48,25	1,375	1873,327964	28,173
Z5	6	0	6	l	4,500	-0,017	54,75	7,875	1789,816487	-55,338

box	year	justorder	order	lightness	centered lightness	egg volume	centered volume
A4	11	1	f	32,75	-1,15	1940,994281	74,68370599
A4	11	2	c	34,75	0,85	1789,078613	-77,2319621
A4	11	3	c	31,5	-2,4	1812,20633	-54,10424444
A4	11	4	c	32	-1,9	1877,492682	11,1821071
A4	11	5	l	38,5	4,6	1911,780968	45,47039344
A5	11	1	f	46,5	-0,4	1631,017701	-93,90109525
A5	11	2	c	44,25	-2,65	1732,854946	7,936149984
A5	11	3	c	44,5	-2,4	1791,022598	66,10380167
A5	11	4	c	46,25	-0,65	1717,362565	-7,556231196
A5	11	5	l	53	6,1	1752,336171	27,41737478
A5a	10	1	f	45,25	4	2106,294776	67,89136861
A5a	10	2	c	40	-1,25	2028,318751	-10,08465605
A5a	10	3	c	39,75	-1,5	1989,5022	-48,9012074
A5a	10	4	c	38,5	-2,75	2026,194857	-12,20855003
A5a	10	5	l	42,75	1,5	2041,706452	3,303044886
B1	11	1	f	34,5	-2,6	2083,099428	-50,10664626
B1	11	2	c	33,75	-3,35	2196,901727	63,69565338
B1	11	3	c	32	-5,1	2095,385637	-37,8204372
B1	11	4	c	34,25	-2,85	2173,23648	40,03040592
B1	11	5	l	51	13,9	2117,407098	-15,79897584
B1a	10	1	f	53,5	0,4	1870,262496	-0,143141802
B1a	10	2	c	51	-2,1	1855,924043	-14,48159494
B1a	10	3	c	52,25	-0,85	1855,602696	-14,80294135
B1a	10	4	c	50,75	-2,35	1878,600632	8,194994058
B1a	10	5	l	58	4,9	1891,638321	21,23268404
B2	11	1	f	25,5	-7,041666667	2066,11936	162,7748491
B2	11	2	c	23,5	-9,041666667	2008,340087	104,9955767
B2	11	3	c	27,25	-5,291666667	1984,245525	80,90101431
B2	11	4	c	29,25	-3,291666667	1894,838072	-8,506439005
B2	11	5	c	38,75	6,208333333	1651,447969	-251,896542
B2	11	6	l	51	18,45833333	1815,076052	-88,26845919
B4	11	1	f	44,5	1,9	1888,078153	33,47068514
B4	11	2	c	38,25	-4,35	1883,669953	29,06248535
B4	11	3	c	39,25	-3,35	1829,858243	-24,74922421
B4	11	4	c	41,25	-1,35	1844,95244	-9,655027566
B4	11	5	l	49,75	7,15	1826,478549	-28,12891873
B4a	10	1	f	37,75	-1,8	2170,414909	58,40545439
B4a	10	2	c	37,25	-2,3	2139,159439	27,14998403
B4a	10	3	c	38,5	-1,05	2080,463969	-31,54548553
B4a	10	4	c	37,75	-1,8	2160,690557	48,68110181
B4a	10	5	l	46,5	6,95	2009,3184	-102,6910547
C1	11	1	f	43	5,05	1849,386242	-173,9473205
C1	11	2	c	34,75	-3,2	1973,676401	-49,65716204
C1	11	3	c	36	-1,95	2138,393198	115,0596351
C1	11	4	c	34,75	-3,2	2110,080475	86,74691215
C1	11	5	l	41,25	3,3	2045,131498	21,7979353
C4	10	1	f	46,25	-2,45	1933,762332	-11,70066623
C4	10	2	c	43,5	-5,2	1911,587942	-33,87505721
C4	10	3	c	45	-3,7	2088,10542	142,6424208
C4	10	4	c	44,75	-3,95	1919,874704	-25,58829467
C4	10	5	l	64	15,3	1873,984596	-71,47840271
E1	11	1	f	38	1,25	1835,550286	-53,84573123
E1	11	2	c	34,25	-2,5	1823,048507	-66,34751016
E1	11	3	c	34	-2,75	1909,230177	19,83416001
E1	11	4	c	39,75	3	1948,306145	58,91012797
E1	11	5	c	27,5	-9,25	1952,710103	63,31408608
E1	11	6	l	47	10,25	1867,530885	-21,86513267
E5	10	1	f	49	2,65	2033,509885	-85,47106718
E5	10	2	c	41,5	-4,85	2108,225572	-10,7553797
E5	10	3	c	43,25	-3,1	2164,737381	45,75642898
E5	10	4	c	43	-3,35	2176,922591	57,94163866
E5	10	5	l	55	8,65	2111,509331	-7,471620768
E6	11	1	f	31,25	-4,35	2196,732717	160,6282192
E6	11	2	c	33,75	-1,85	2074,290352	38,18585455
E6	11	3	c	35,5	-0,1	2028,514319	-7,590178734
E6	11	4	c	32,75	-2,85	1966,505165	-69,59933249
E6	11	5	l	44,75	9,15	1914,479935	-121,6245625
G2b	10	1	f	46,75	2,2	1744,514525	-6,07082784
G2b	10	2	c	43,5	-1,05	1749,154461	-1,4308917
G2b	10	3	c	41,25	-3,3	1782,924123	32,33876952
G2b	10	4	c	40,75	-3,8	1729,733646	-20,851707
G2b	10	5	l	50,5	5,95	1746,60001	-3,98534298

H6	11	1 f	51,25	4,25	2083,430325	116,0474806
H6	11	2 c	45,25	-1,75	1830,314661	-137,0681836
H6	11	3 c	44	-3	1966,822674	-0,560169924
H6	11	4 c	39,75	-7,25	1989,151688	21,76884388
H6	11	5 l	54,75	7,75	1967,194874	-0,187970904
I1	11	1 f	26	-1,6875	1799,504524	-17,6721528
I1	11	2 c	22,25	-5,4375	1795,842829	-21,33384825
I1	11	3 c	25,5	-2,1875	1812,132685	-5,04399231
I1	11	4 l	37	9,3125	1861,226671	44,04999336
I1a	10	1 f	47,75	-5,125	1987,921829	90,72772967
I1a	10	2 c	54,25	1,375	1692,747312	-204,446788
I1a	10	3 c	53,75	0,875	1955,769179	58,57507955
I1a	10	4 c	50,25	-2,625	1951,388507	54,19440752
I1a	10	5 c	54,5	1,625	1929,945022	32,75092203
I1a	10	6 l	56,75	3,875	1865,392749	-31,80135082
I12	11	1 f	32,5	-2,3	1674,884377	-191,7254707
I12	11	2 c	31	-3,8	1830,894607	-35,71524053
I12	11	3 c	31,75	-3,05	1982,39599	115,7861423
I12	11	4 c	35	0,2	1960,0618	93,45195304
I12	11	5 l	43,75	8,95	1884,812463	18,20261584
I18	11	1 f	44,5	-0,0625	1883,868851	-50,16840216
I18	11	2 c	36	-8,5625	1876,041948	-57,99530484
I18	11	3 c	35,75	-8,8125	1910,685024	-23,35222884
I18	11	4 l	62	17,4375	2065,553189	131,5159358
I18a	10	1 f	49,75	0,65	2011,852237	-14,85410904
I18a	10	2 c	47	-2,1	2155,629894	128,9235482
I18a	10	3 c	47,5	-1,6	1977,940195	-48,76615104
I18a	10	4 c	45	-4,1	1972,520338	-54,18600825
I18a	10	5 l	56,25	7,15	2015,589066	-11,11727988
I19	10	1 f	72,75	3,708333333	1560,039	-230,4705586
I19	10	2 c	67,5	-1,541666667	1787,181498	-3,328060675
I19	10	3 c	66,25	-2,791666667	1839,027621	48,51806247
I19	10	4 c	67,25	-1,791666667	1841,949805	51,44024619
I19	10	5 c	65	-4,041666667	1804,622072	14,11251285
I19	10	6 l	75,5	6,458333333	1910,237356	119,7277978
I22	11	1 f	32,25	-4,4	1638,308829	-184,6553905
I22	11	2 c	32	-4,65	1843,420318	20,45609888
I22	11	3 c	34	-2,65	1847,766043	24,80182367
I22	11	4 c	34	-2,65	1913,231776	90,267557
I22	11	5 l	51	14,35	1872,09413	49,12991099
I7	10	1 f	43,75	-2,25	1745,564403	-80,50396655
I7	10	2 c	40	-6	1845,658066	19,5896968
I7	10	3 c	44,25	-1,75	1855,941126	29,87275693
I7	10	4 c	43,25	-2,75	1840,284765	14,21639545
I7	10	5 l	58,75	12,75	1842,893487	16,82511736
I8	11	1 f	48	1,55	1903,052968	-25,89299366
I8	11	2 c	40,75	-5,7	1889,273523	-39,67243886
I8	11	3 c	43,25	-3,2	1946,892386	17,94642427
I8	11	4 c	45,75	-0,7	1967,921802	38,97584026
I8	11	5 l	54,5	8,05	1937,58913	8,643167982
J21	11	1 f	31,75	-2,875	1863,056704	-19,20804763
J21	11	2 c	33	-1,625	1856,272561	-25,99219055
J21	11	3 c	31,75	-2,875	1930,650556	48,385804
J21	11	4 l	42	7,375	1879,079186	-3,185565825
J28	11	1 f	46,25	0,9	1697,61915	-5,08231524
J28	11	2 c	36,75	-8,6	1705,650242	2,94877716
J28	11	3 c	50	4,65	1742,050632	39,34916628
J28	11	4 c	44,25	-1,1	1726,833333	24,13186788
J28	11	5 l	49,5	4,15	1641,353969	-61,34749608
J28a	10	1 f	42	-0,15	1802,983818	-27,43282126
J28a	10	2 c	38,5	-3,65	1774,27838	-56,13825898
J28a	10	3 c	36,5	-5,65	1949,286708	118,8700689
J28a	10	4 c	38,5	-3,65	1842,646575	12,22993586
J28a	10	5 l	55,25	13,1	1782,887715	-47,5289245
J29	11	1 f	29,25	-4,916666667	1990,912884	31,7645538
J29	11	2 c	31	-3,166666667	1974,924956	15,77662654
J29	11	3 c	32,75	-1,416666667	1986,722595	27,57426529
J29	11	4 c	36,5	2,333333333	1969,31706	10,16873028
J29	11	5 c	39,25	5,083333333	1911,587369	-47,56096044
J29	11	6 l	36,25	2,083333333	1921,425114	-37,72321547
J34	11	1 f	35	3,05	1815,540089	-63,96504997
J34	11	2 c	27,5	-4,45	1892,075456	12,57031751
J34	11	3 c	27,75	-4,2	1946,792412	67,2872735
J34	11	4 c	28,75	-3,2	1897,970508	18,46536926

J34	11	5 l	40,75	8,8	1845,147228	-34,35791032
J36	11	1 f	40,5	-3,625	2302,75562	206,7526086
J36	11	2 c	43	-1,125	2090,391384	-5,611627537
J36	11	3 c	43	-1,125	2026,5721	-69,43091155
J36	11	4 l	50	5,875	1964,292942	-131,7100695
J37	11	1 f	38,75	1,5	1757,680846	32,62771726
J37	11	2 c	34,5	-2,75	1651,420457	-73,63267178
J37	11	3 c	34,75	-2,5	1673,298104	-51,75502481
J37	11	4 c	34,5	-2,75	1762,259712	37,20658294
J37	11	5 l	43,75	6,5	1780,606525	55,55339638
J38	11	1 f	27,25	-3	2042,330904	-145,372594
J38	11	2 c	28,5	-1,75	2053,406625	-134,296873
J38	11	3 c	31	0,75	2284,284951	96,58145298
J38	11	4 c	31,25	1	2315,876577	128,1730786
J38	11	5 l	33,25	3	2242,618433	54,91493544
J41	11	1 f	39,25	-0,85	1959,847947	141,9411729
J41	11	2 c	36,25	-3,85	1930,276331	112,3695562
J41	11	3 c	38,75	-1,35	1889,3966	71,48982589
J41	11	4 c	39	-1,1	1756,484972	-61,42180247
J41	11	5 l	47,25	7,15	1553,528022	-264,3787525
J42	11	1 f	40,75	4,2	2051,372971	36,76873213
J42	11	2 c	34,25	-2,3	1921,489797	-93,11444279
J42	11	3 c	31,75	-4,8	2028,542063	13,93782325
J42	11	4 c	33,5	-3,05	2016,787483	2,183243292
J42	11	5 l	42,5	5,95	2054,828883	40,22464411
J42a	10	1 f	49	-5,8	1511,423539	-137,9257036
J42a	10	2 c	50,5	-4,3	1662,231313	12,88207062
J42a	10	3 c	50,25	-4,55	1746,449724	97,10048202
J42a	10	4 c	54,25	-0,55	1741,849781	92,50053906
J42a	10	5 l	70	15,2	1584,791854	-64,55738814
J44	11	1 f	23,5	-5,45	2027,217435	66,81570404
J44	11	2 c	30	1,05	2014,903716	54,50198456
J44	11	3 c	22,5	-6,45	1933,95577	-26,44596157
J44	11	4 c	25,75	-3,2	1961,304531	0,902799144
J44	11	5 l	43	14,05	1864,627205	-95,77452619
J46	11	1 f	36,25	0,6	1959,113366	21,50638084
J46	11	2 c	34	-1,65	1915,328607	-22,27837733
J46	11	3 c	29,5	-6,15	1966,942658	29,33567338
J46	11	4 c	32,5	-3,15	1975,16893	37,56194482
J46	11	5 l	46	10,35	1871,481363	-66,12562172
J8	11	1 f	42,5	2,1875	2041,224184	71,44647464
J8	11	2 c	36,75	-3,5625	1881,073239	-88,70446996
J8	11	3 c	35,5	-4,8125	1994,001455	24,22374629
J8	11	4 l	46,5	6,1875	1962,811958	-6,965750977
K2	11	1 f	40,25	-1,55	2295,378932	74,31311939
K2	11	2 c	37,75	-4,05	2250,894094	29,82828218
K2	11	3 c	38,75	-3,05	2192,4989	-28,56691264
K2	11	4 c	41,75	-0,05	2235,731374	14,66556194
K2	11	5 l	50,5	8,7	2130,825761	-90,24005086
K21	11	1 f	38,5	-1	2178,431229	16,71693637
K21	11	2 c	35,25	-4,25	2105,215246	-56,49904664
K21	11	3 c	38,25	-1,25	2155,513375	-6,200917824
K21	11	4 c	39,75	0,25	2128,181439	-33,53285363
K21	11	5 l	45,75	6,25	2241,230174	79,51588174
M3	11	1 f	34,5	-5,65	1966,930457	47,55166978
M3	11	2 c	37,25	-2,9	1940,008125	20,62933792
M3	11	3 c	38,25	-1,9	1896,875242	-22,50354488
M3	11	4 c	39,25	-0,9	1842,481563	-76,89722411
M3	11	5 l	51,5	11,35	1950,598548	31,21976128
O1	11	1 f	39,75	-2,05	2095,968804	36,94741186
O1	11	2 c	44	2,2	2054,82669	-4,194701244
O1	11	3 c	41,75	-0,05	1985,550996	-73,47039518
O1	11	4 c	37,5	-4,3	2106,36701	47,34561826
O1	11	5 l	46	4,2	2052,393458	-6,627933684
O16	11	1 f	41	-1,8	1681,60443	-42,38592456
O16	11	2 c	38,75	-4,05	1740,206039	16,21568409
O16	11	3 c	40,25	-2,55	1739,169228	15,17887347
O16	11	4 c	38,5	-4,3	1746,313052	22,32269745
O16	11	5 l	55,5	12,7	1712,659025	-11,33133045
O20	11	1 f	29	-4,25	2184,553266	-10,27541095
O20	11	2 c	33,75	0,5	2208,345726	13,51704938
O20	11	3 c	28,25	-5	2262,146175	67,31749865
O20	11	4 c	32,25	-1	2216,592151	21,76347449
O20	11	5 l	43	9,75	2102,506065	-92,32261159

P14	11	1 f	41,75	-1,65	1915,978459	84,40052638
P14	11	2 c	37,5	-5,9	1848,256378	16,67844493
P14	11	3 c	36,25	-7,15	1793,491363	-38,08656938
P14	11	4 c	37	-6,4	1802,699553	-28,87837964
P14	11	5 l	64,5	21,1	1797,46391	-34,1140223
P4	11	1 f	25,25	-4,45	1988,449102	38,55781805
P4	11	2 c	27,75	-1,95	1961,887331	11,99604701
P4	11	3 c	29,5	-0,2	2030,442718	80,55143429
P4	11	4 c	28,25	-1,45	1935,887209	-14,00407531
P4	11	5 l	37,75	8,05	1832,79006	-117,101224
P9	11	1 f	24,5	-3,4375	2018,603101	-39,92258593
P9	11	2 c	22	-5,9375	2089,691136	31,16544911
P9	11	3 c	26	-1,9375	2102,12554	43,59985346
P9	11	4 l	39,25	11,3125	2023,68297	-34,84271665
Q4	10	1 f	28,75	-5,05	1877,218572	-90,06119723
Q4	10	2 c	29	-4,8	1995,20148	27,92171062
Q4	10	3 c	31,5	-2,3	2030,201733	62,92196359
Q4	10	4 c	35,25	1,45	2019,505477	52,22570758
Q4	10	5 l	44,5	10,7	1914,271585	-53,00818457
U13	11	1 f	37,5	-1,55	1726,582203	-38,1685835
U13	11	2 c	33,5	-5,55	1767,554159	2,803372182
U13	11	3 c	35,25	-3,8	1816,59531	51,84452338
U13	11	4 c	38	-1,05	1769,323145	4,572358182
U13	11	5 l	51	11,95	1743,699116	-21,05167025
U2	11	1 f	44,75	1,75	1820,445	-80,45480673
U2	11	2 c	34,25	-8,75	1863,240144	-37,65966225
U2	11	3 c	40,75	-2,25	1973,516053	72,61624596
U2	11	4 c	45,5	2,5	1939,210128	38,31032127
U2	11	5 l	49,75	6,75	1908,087708	7,18790175
U8	11	1 f	34	-3,1	1784,466005	-82,12670627
U8	11	2 c	31,25	-5,85	1857,228375	-9,364336134
U8	11	3 c	34,75	-2,35	1959,733968	93,14125696
U8	11	4 c	37,5	0,4	1919,73333	53,14061923
U8	11	5 l	48	10,9	1811,801877	-54,79083377
U9	11	1 f	29,25	-3,9	2062,836835	102,3761118
U9	11	2 c	29,25	-3,9	1970,345424	9,884700762
U9	11	3 c	32	-1,15	1943,839613	-16,62111053
U9	11	4 c	34,5	1,35	1905,707514	-54,75320924
U9	11	5 l	40,75	7,6	1919,57423	-40,88649284
V1	11	1 f	35,5	-5,35	2084,86739	-90,15442054
V1	11	2 c	37,25	-3,6	2083,099428	-91,92238297
V1	11	3 c	38,75	-2,1	2190,564436	15,54262556
V1	11	4 c	37,5	-3,35	2224,149274	49,12746278
V1	11	5 l	55,25	14,4	2292,428526	117,4067152
V9	11	1 f	44,75	-1,4375	2162,66112	87,48652251
V9	11	2 c	43,5	-2,6875	2097,269889	22,09529151
V9	11	3 c	44,25	-1,9375	2051,888116	-23,28648117
V9	11	4 l	52,25	6,0625	1988,879265	-86,29533285
W1	10	1 f	57	7	1473,344035	-152,9920273
W1	10	2 c	47	-3	1513,339331	-112,9967308
W1	10	3 c	46,25	-3,75	1704,815704	78,47964242
W1	10	4 c	44,5	-5,5	1730,818383	104,4823214
W1	10	5 l	55,25	5,25	1709,362856	83,02679426
W2	11	1 f	34,5	-3,1	1940,156025	17,48059721
W2	11	2 c	31,25	-6,35	2002,686236	80,01080777
W2	11	3 c	38	0,4	2015,488682	92,81325413
W2	11	4 c	33,75	-3,85	1919,232	-3,443427792
W2	11	5 l	50,5	12,9	1735,814196	-186,8612313
W4	11	1 f	44	-0,25	1983,33474	34,6944942
W4	11	2 c	43,75	-0,5	2016,890032	68,24978559
W4	11	3 c	39	-5,25	2020,201943	71,56169703
W4	11	4 c	41,5	-2,75	1877,174633	-71,46561354
W4	11	5 l	53	8,75	1845,599883	-103,0403633
W5	11	1 f	28,5	0,416666667	2064,829923	-146,1424297
W5	11	2 c	24,25	-3,833333333	2145,053835	-65,91851728
W5	11	3 c	26,75	-1,333333333	2265,583698	54,61134587
W5	11	4 c	30	1,916666667	2318,267571	107,2952185
W5	11	5 c	23	-5,083333333	2258,840745	47,8683926
W5	11	6 l	36	7,916666667	2213,258342	2,28599
W7	11	1 f	30	-3,9	1922,349184	33,74493163
W7	11	2 c	31,75	-2,15	1876,041948	-12,56230388
W7	11	3 c	31,25	-2,65	1904,157559	15,5533066
W7	11	4 c	33	-0,9	1871,929597	-16,67465471
W7	11	5 l	43,5	9,6	1868,542973	-20,06127962

X	11	1	f	39,5	-1,541666667	2250,404478	48,05167206
X	11	2	c	38,75	-2,291666667	2247,613248	45,26044207
X	11	3	c	40,5	-0,541666667	2278,812282	76,45947582
X	11	4	c	40,75	-0,291666667	2028,994748	-173,3580577
X	11	5	c	41,75	0,708333333	2230,501555	28,14874866
X	11	6	l	45	3,958333333	2177,790525	-24,56228094
Y1	10	1	f	48,5	4,3	1760,200192	-143,0876226
Y1	10	2	c	42,5	-1,7	1882,860377	-20,42743739
Y1	10	3	c	38,75	-5,45	1984,875904	81,58809007
Y1	10	4	c	42,5	-1,7	1998,639549	95,35173445
Y1	10	5	l	48,75	4,55	1889,86305	-13,42476458
Y6	11	1	f	36,5	-8,5	2110,824826	37,44761615
Y6	11	2	c	41,5	-3,5	2184,471313	111,0941034
Y6	11	3	c	38,75	-6,25	2118,991999	45,6147893
Y6	11	4	c	50,75	5,75	2033,509885	-39,86732458
Y6	11	5	c	44,5	-0,5	2032,617287	-40,75992199
Y6	11	6	l	58	13	1959,847947	-113,5292623
Y6a	11	1	f	37,25	-4,05	2086,518528	216,8157461
Y6a	11	2	c	42,5	1,2	1916,001193	46,29841098
Y6a	11	3	c	40,75	-0,55	1843,629141	-26,07364086
Y6a	11	4	c	41,5	0,2	1837,361809	-32,34097272
Y6a	11	5	l	44,5	3,2	1665,003238	-204,6995435
Z7	10	1	f	47	1,5	2029,004139	178,2620034
Z7	10	2	c	47	1,5	1873,112624	22,37048802
Z7	10	3	c	40,75	-4,75	1805,693756	-45,04837956
Z7	10	4	c	39,5	-6	1794,714173	-56,0279625
Z7	10	5	l	53,25	7,75	1751,185986	-99,55614936
Z8	10	1	f	36,25	-2,5	1922,1084	-75,59553244
Z8	10	2	c	34,5	-4,25	1959,040329	-38,66360296
Z8	10	3	c	32,25	-6,5	2036,598202	38,89426964
Z8	10	4	c	36,5	-2,25	2055,429815	57,72588296
Z8	10	5	l	54,25	15,5	2015,342915	17,63898281
Z9	11	1	f	35	-1,3	2063,92339	124,4683369
Z9	11	2	c	34,25	-2,05	1998,744188	59,28913435
Z9	11	3	c	32,75	-3,55	1855,554356	-83,9006969
Z9	11	4	c	36	-0,3	1880,311974	-59,14307891
Z9	11	5	l	43,5	7,2	1898,741358	-40,71369545
